# Assisted Robots in Therapies for Children with Autism in Early Childhood

**DOI:** 10.3390/s24051503

**Published:** 2024-02-26

**Authors:** Ana Gómez-Espinosa, José Carlos Moreno, Sagrario Pérez-de la Cruz

**Affiliations:** 1 Department of Informatics, University of Almería, ceiA3, CIESOL, 04120 Almería, Spain; age597@ual.es; 2 Department of Nursing, Physical Therapy and Medicine, University of Almería, 04120 Almería, Spain; spd205@ual.es

**Keywords:** child, early ages, autism, robot, ADS, social robots

## Abstract

Children with autism spectrum disorder (ASD) have deficits that affect their social relationships, communication, and flexibility in reasoning. There are different types of treatment (pharmacological, educational, psychological, and rehabilitative). Currently, one way to address this problem is by using robotic systems to address the abilities that are altered in these children. The aim of this review will be to analyse the effectiveness of the incorporation of the different robotic systems currently existing in the treatment of children up to 10 years of age diagnosed with autism. A systematic review has been carried out in the PubMed, Scopus, Web of Science, and Dialnet databases, with the following descriptors: child, autism, and robot. The search yielded 578 papers, and nine were selected after the application of the PRISMA guideline. The quality of the studies was analysed with the PEDRo scale, and only those with a score between four and six were selected. From this study, the conclusion is that the use of robots, in general, improves children’s behaviour in the short term, but longer-term experiences are necessary to achieve more conclusive results.

## 1. Introduction

According to the World Health Organisation (WHO), it is estimated that 1 in 160 children has autism spectrum disorder (ASD) [1], but other studies suggest that this figure is even higher, reaching up to 1 per 100 children [2]. This estimate represents an average figure, as the observed prevalence varies considerably between studies [1]. Furthermore, Yang et al. [3] studied different databases to conduct a sociodemographic study and observed that there was a high age-standardised prevalence of ASD in the under-5 subgroup, in the subgroup of high sociodemographic index (SDI), and in the subgroup of high-income North America, respectively. Each subgroup is a smaller group represented by a common characteristic. In the case of the under-5 group, all patients are under five years of age; the North American group only comprises autistic people from that area; and the high-SDI group comprises autistic people who live in areas with a favourable socio-economic level and relatively good access to resources and services, that is, they live in families with relatively high levels of income and education. There are many possible explanations for this apparent increase in prevalence, including increased awareness, expanded diagnostic criteria, better diagnostic tools, and improved communication [1,2,3].

Autism is a complex developmental disorder that manifests itself in behaviours at the level of social and communicative interaction. Currently, through early care techniques and therapy during the first years of life, people with autism can achieve an autonomous life [4]. Therefore, ASD is a significant challenge in public health and developmental psychology, which affects an increasing number of children around the world. Children with ASD have a wide range of symptoms and needs, from difficulties in communication and social interaction to repetitive patterns of behaviour [4]. As the incidence of ASD continues to increase, it has become imperative to develop effective and accessible interventions that can improve the quality of life of these children and their families. In this context, robot-assisted therapies have emerged as a promising frontier in the care and treatment support of autistic children. The convergence of advanced technologies, such as robotics, artificial intelligence, and developmental psychology, has enabled the creation of specially designed robots to interact with children with ASD, which are robots that possess unique characteristics that make them ideal for these interventions [5]. They can offer repeatable and predictable interactions, which is especially beneficial for children who often find social situations unpredictable and overwhelming. In addition, these robots can adapt to the individual needs of the children, thus personalising therapy effectively and efficiently. They also eliminate the fear of social criticism often experienced by autistic children, creating a more comfortable and safe learning environment.

The research of various authors converges on specific thematic areas, reflecting the diversity and richness of investigations on the use of robots for children with ASD. In the field of robot-assisted intervention development, refs. [6,7,8,9,10,11] address the need for personalised interventions. From attention assessment to influencing gaze and joint attention, these authors seek to provide long-term therapies in home environments.

The application of machine learning and deep learning, subfields of artificial intelligence that focus on developing algorithms and models capable of learning patterns and performing specific tasks without the need to be explicitly programmed, in ASD classification and robotic therapies is a shared concern for researchers [12,13,14,15,16]. These works focus on the use of advanced algorithms and techniques to improve diagnostic accuracy and gain a better understanding of the patterns associated with the disorder. Another use, also related to this field, is Mihalache et al.’s [17] research on gaze perception in children with ASD that sheds light on how head and pupil rotations in 2D and 3D impact typical development and the effects of the disorder.

The implementation of robots in assisted therapy and rehabilitation for children with ASD is a prominent research area for authors such as [18,19,20,21,22,23,24,25,26,27,28]. These researchers explore everything from robot autonomy in non-invasive therapies to programming for specific skill improvements. Some authors better describe how therapy was focused or what its approach was, as in the case of [29] focussing on the development of a robotic music therapy platform, demonstrating the innovative application of non-conventional interventions, or [30,31] using social stories to promote social communication in children with ASD, using a humanoid robot to contribute to improving communication skills and social interaction.

Designing social robots and emotional communication are central points for authors, such as [30,32,33,34,35,36,37,38,39]. Their work focuses on developing robots with social and emotional abilities to achieve effective communication with autistic children. Redesigns or improvements of robots with other uses were also found, as in the case of [40] where an existing robot used as a commercial toy was modified for use as a therapy robot by adding the necessary software and hardware.

Other studies performed reviews, although they were not systematic reviews focusing solely on the use of robots at early ages for autistic children. Some of these articles were mainly aimed at showing the robots used in the articles [41] and others had samples with children of non-early ages, adolescents, or adults [21].

Finally, articles were found that did not perform robotic therapy but provided advanced insights into the technologies used (algorithms) in therapies for autistic children from different articles [42], the ethical and social aspects of robot-assisted therapy [43], and the socio-emotional outcomes of robot-assisted therapy [44], providing valuable information on the expectations and ethical acceptability of this new form of intervention. 

Despite the growing interest and excitement surrounding robot-assisted therapies in the context of ASD at an early age, there is a critical need to evaluate and synthesise the existing research to better understand the current state of the field and its efficacy. This review of the literature aims to address this need by analysing and summarising the relevant scientific literature related to the use of robots in therapies for autistic children. We will explore the different intervention modalities, ranging from the improvement of communication skills to the promotion of social and emotional skills. In addition, we will examine the results obtained in previous studies, identifying trends and patterns that have emerged in the research.

The ultimate goal of this review is to shed light on the possibilities and limitations of robot-assisted therapies in the treatment of children with ASD at early ages (children 0–6 years old [45]), although in some of the reviewed articles, some of the children do not fall within this age range, due to the difficulty of finding articles with homogeneous groups of children with autism (similar ages, depth of the disorder, etc.). Interest in this age range is motivated by action programmes such as “early treatment” in countries such as Spain. These programmes provide care for children aged 0–6 years with permanent and/or temporary disorders and their families. Some elements of autism show alterations in very early ages, but issues such as language and social behaviour, from normal development, can be prolonged over time. That is why the period extends from 0 to 6 years of age. For example, around six years old, children use conjunctions and extend the verb tenses (language). From the age of 4, children start with the ties of friendship, but with egocentric features. The parallel game evolves to a symbolic, cooperative level, and one with rules. They express themselves emotionally with their peers and are influenced by them [46]. 

**Figure 1:**
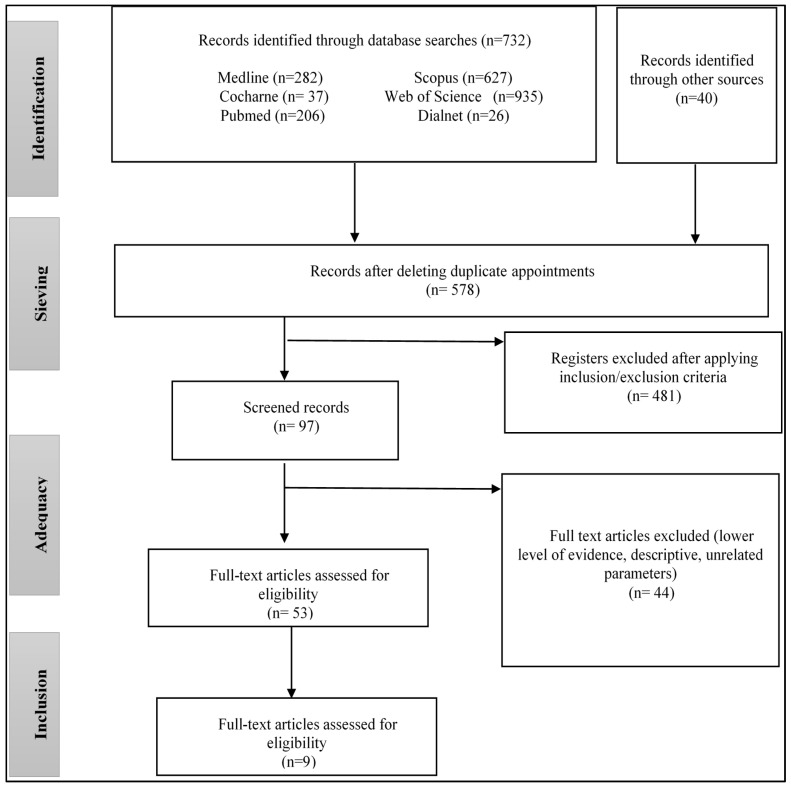
PRISMA flow diagram for reviewed studies.

We also aim to understand how these new therapies, under the scope of human–computer interaction, compare with traditional therapy approaches, as well as what evidence supports their efficacy and efficiency in improving outcomes for autistic children. In addition, we will explore the clinical implications of these therapies and future research directions in this exciting interdisciplinary field that combines advanced technology with attention to the education, mental health, and well-being of autistic children. To achieve this throughout this work, a large number of articles have been reviewed, as can be seen in Figure 1. 

In summary, this review of the literature aims to contribute to current knowledge about therapies for autistic children in early childhood and to highlight the promising role that robots can play in the treatment of this complex and heterogeneous disorder. As we advance the understanding and application of these therapies, we hope to provide a clearer picture of how robots can be effectively integrated into the care of these children, thus improving their lives and development. Furthermore, these collective works reflect the commitment of the scientific community to address various aspects of the use of robots to enhance the lives of children with ASD, from personalised interventions and advanced machine learning applications to ethical considerations in the implementation and design of social and emotional robots.

## 2. Materials and Methods

A literature search was conducted between May and August 2023 in the Scopus, Web of Science, Dialnet, Pubmed, and Cochrane databases with the following search strategy: “child” [Abstract] AND “autism” [Abstract] AND “robot” [Abstract]. It was decided to choose only these three keywords to cover the maximum number of papers, taking into account that the main interest is to analyse the effect of robots in therapies with autistic children at an early age.

For this review, randomised and nonrandomised clinical trials, literature reviews, and descriptive and case studies, which did not describe, or only partially described, the type of therapy used (cognitive, motor, social skills, etc.) and the method of therapy (time used, number of patients, number of sessions, etc.), were excluded. Furthermore, we also excluded trials or tests with samples that did not include young children or included children older than 10 years of age in the samples, despite including young children, as shown in Table 1. Furthermore, electronic journals considered to be of greatest relevance to the subject under study were searched handheld. Searches based on the snowball technique were also carried out, reviewing the reference lists of articles already included for review in this work to verify the existence of additional non-emerging articles in databases. The age exclusion criterion was found to be the most relevant, eliminating some potential articles such as [7,8,9,17,19,20,23,24,25,28,29,32,35], which clearly showed the therapies performed, the robots used, and the relevant results provided.

The PRISMA2020 recommendations [47] were followed to perform an exhaustive review. This guideline has been widely endorsed and adopted [48]. The PRISMA identification study flow diagram is shown in Figure 1. The titles and abstracts were selected, excluding duplicate articles and those that did not meet the selection criteria. The following data were extracted from each study: author and date of publication, type of study, number of participants, rating scales, participants (if the number of males/females was specified, it was added too), intervention, methodology, and results obtained.

Methodological quality assessment was performed using the PEDRo scale [49]. For studies selected in Table 2. Table 3 shows the score on this scale. It has 11 items, and each category is scored with one point if it meets the requirements. Therefore, the higher the score, the higher the methodological quality of the article, considering that a study with a score ≥ 6 has a high quality level (6–8: good; 9–10: excellent), and a study with a score ≤ 5 has a low level (4–5: acceptable; <4: poor).

## 3. Results

A total of 9 studies of 578 potentials met the selection criteria in Figure 1. The most relevant information on the articles studied for this review can be found in Table 2, where the author, the type of article, the sample, the intervention, the type of scale used, and the results of each work are indicated. There are six study cases [10,11,12,13,14,15], two pilot studies [16,17], and one randomised controlled trial (RCT) [50]. In these papers, the number of subjects included was 186, of which 27 were known to be female. Although the sex of all groups is not indicated, it is well known that the ratio of autistic males is higher than that of females [3,51]. The sample sizes were very disparate, ranging from experimental groups of 6 to 35 children, depending on the location where the tests were conducted and the ages of the children.

**Table 2:** Selection of studies.

Authors	Type of Study	Sample (Total of Males) and Age	Intervention	Scale and Evaluation Methodology	Result
Pop et al. [37]	Case study	N = 20 Ages = 4 to 9 years	Robot Probo. CG (*n* = 7); G1 computer-presented social stories (*n* = 6); G2 robot-assisted therapy (*n* = 7). It was used as a story-telling agent.	Asking questions, eye gaze, asking for help, and greeting	The use of the social robot to implement a social story intervention was more effective in improving the independence to express social skills in participants than the computer screen.
Wainer et al. [52]	Case study	N = 6 (1 female) Ages = 8.5 ± 0.55 years	Robot KASPAR. Imitative and collaborative games with autistic children accompanied by a partner with and without the presence of the robot. ABAB, ‘A’–interacting with a human. adult, and ‘B’ interacting with the robot KASPAR. 20 sessions.	Promoting to choose, urging to comply, other forms of talking, successful shape selection, pose, gaze, and gaze shift, positive affect	The data did not show that KASPAR increased the persistence of a positive affect in other children, but showed that the duration of positive affect when looking at other children was longer.
Dehkordi et al. [40]	Case study	ADS: N = 35 Ages = 4.7 ± 2.56 years Normal: N = 16, Ages = 4.5 ± 2.2 years	Parrot robot. The robot was used to talk, sing, move, and react to interactions to capture the children’s attention. The experiment was carried out in two modes: (a) individual interaction mode (this therapy had a duration of 8 to 12 min) and (b) group interaction mode (in group of six children with 20 to 30 min of therapy).	The evaluation was carried out with videos that were observed by an expert. Different parameters were evaluated: eye contact or if the duration was less than 3 s, smile, physical proximity to the robot, gaze or pointing, attention, etc.	The robot could encourage children to interact according to their preferences that match the three functionalities of the robot (verbal, functional, and perceptual).
Boccanfuso et al. [38]	Pilot study	EG: N = 8 Ages = 3 to 6 years CG: N = 3 aged = 3 to 6 years	Robot Charlie. The robot interacted with the children through various interactive games and activities. The interaction involved the robot responding to the children’s actions, providing positive sensory feedback, and promoting engagement and trust through fun and social exchanges. The intervention divided into three parts: one hour of speech therapy per week for 6 weeks (MLSUD provides a total meaningful spoken language score during the evaluation period of 1.0–1.5 h), two 30-min sessions per week for a total of 6 weeks, or 12 total intervention sessions with the robot.	(1) VABS-II Communication Domain, (2) VABS-II socialisation domain, (3) VABS-II Receptive and Expressive Communication v-scale scores, (4) MLSUD, (5) UIA social imitation, (6) UIA requesting (7) UIA joint attention, (8) MIS and (9) EVT2	The results within the group showed an increase in social interaction skills, as reported by caregivers on the Vineland II Parent/Caregiver Rating Form. Increases between groups in the Receptive Language and Play and Leisure scales.
David et al. [53]	Case study	N = 5 (1 female) Ages = 4.68 ± 0.81 years	Robot NAO. The interaction involves giving the child an instruction to pay attention to what the robot is looking at, waiting for the child’s response, and providing feedback based on the child’s answer. 1. Baseline measurements (BM) for six to eight measurements, until a stable baseline level has been established. 2. Robot-enhanced treatment (RET) for 8 sessions. 3. Standard human treatment (SHT) for 8 sessions. 4. RET or SHT, depending on which of the treatments worked best for each child, for 4 sessions. Each session lasted 10 min each day.	Head orientation, pointing, relevant verbalisation (vocal instructions that are in the context of the experiments/tasks being implemented) and delays in performing such behaviours.	Children kept their interest throughout the sessions, showing great adherence to the treatment and improving their joint attention skills.
So et al. [54]	Case study	N = 45 ADS *n* = 15 (6 females) Ages= 5.83 ± 0.83 years CG: ADS *n* = 15 (6 females): Ages = 5.67 ± 0.35 years TD: *n* = 15 (6 females) Ages = 5.33 ± 0.67 years	Robot NAO. EG: received four 30-min robot-based gestural training sessions. The social robot narrated five stories and gestured. Children with ASD were told to mimic gestures during training. CG and TD: children of the same age received gestural training after the completion of the research.	The patients were diagnosed with ADOS test. PEP3, SCQ, BOT, ANT, and gestural recognition task	Children with ASD in the intervention condition were more likely to produce accurate or appropriate intransitive gestures in training and novel stories than those in the wait list control. Positive learning outcomes were maintained in delayed post-tests.
Zhang et al. [55]	Pilot study	ADS: N = 20 (2 females) Ages = 6.79 ± 0.93 years	Robot NAO. The therapy lasted 25 min. Each child participated in a series of tasks in the following order: warm-up session, distrust and deception tasks, and a short interview about their anthropomorphic thinking of the robot.	The different groups were studied with Welch’s *t*-test to compare the distrust and deception tasks, respectively.	This study shows how children with ASD learn to be distrustful. This learning is less than in children with TD.
Zheng et al. [50]	RCT	N = 20 Ages = 1.64 to 3.14 years	Robot NAO. The child interacts through joint attention games, visual tracking of stimuli, and responses to social cues from the robot. Participants were divided into a waiting list control group (WLC; *n* = 11) and an immediate robotic intervention group (RI; *n* = 12). Each group received interventions between 3 and 9 weeks. Duration: 10 min.	After and before intervention measured: prompt level and target hit rate.	Patterns of significant improvement and worsening performance within the system strongly suggest that robotic intervention systems may not be an appropriate additional intervention tool for all young children on the autism spectrum.
Davide et al. [56]	Case study	N = 24 (5 females) Ages = 5.79 ± 1.02 years	Robot Cozmo. The robot stands between two squares of different colours. The child responds verbally or manually. Depending on the response, the robot changes its expression. There were different phases: initiating, responding, and maintaining social interaction, joint attention, and behavioural request. Each training session: 12 turns of a robot game for ten minutes.	The ADOS test is used to measure social interaction, behaviour request, and joint attention. The ESCS evaluates the child’s ability to communicate efficiently, making requests, and responding to the activities proposed by the adult. The results are then statistically compared.	Iteration-based therapies obtained better results than individual therapies.

CG: control group. EG: experimental group. RCT: randomised controlled trial. VABS-II: Vineland Adaptive Behaviour Scale II. SCQ: Social Communication Questionnaire. MLSUD: mean length spontaneous utterance determination. UIA: unstructured imitation assessment. MIS: motor imitation scale. EVT2: motor imitation scale 2. TD: typically developing. ESCS: Early Social Communication Scale.

### 3.1. Methodological Quality

The scores obtained on the PEDRo scale are shown in Table 3. They ranged from four [52,55] to six [37,38,50,53,54] points. Studies that scored less than four points, which means poor quality, were excluded from the review. Of the studies reviewed, 55.56% showed high quality (≥6 points), while 44.44% had a low level (4–5 points considered acceptable). Some of the articles reviewed that met all inclusion criteria but scored less than four points on the PEDRo scale were [11,33,39], which were left out of this review due to the lack of methodological quality.

**Table 3:** Evaluation of the PEDRo scale.

Authors	1	2	3	4	5	6	7	8	9	10	11	Total Score
Pop et al. [37]	X	X	X		X			X		X		6
Wainer et al. [52]	X								X	X	X	4
Dehkordi et al. [40]	X				X	X	X	X				5
Boccanfuso et al. [38]	X			X				X	X	X	X	6
David et al. [53]	X				X	X			X	X	X	6
So et al. [54]	X	X		X				X		X	X	6
Zhang et al. [55]	X			X						X	X	4
Zheng et al. [50]	X	X		X			X			X	X	6
Davide et al. [56]	X	X		X						X	X	5

When the box in the table is marked with an X, the item is evaluated as positive. Items mean the following: (1) Choice criteria were specified. (2) Subjects were randomly assigned to groups (in a crossover study, subjects were randomly assigned as they received treatments). (3) Allocation was concealed. (4) Groups were similar at baseline with respect to the most important prognostic indicators. (5) All subjects were blinded. (6) All therapy-administering therapists were blinded. (7) All assessors measuring at least one key outcome were blinded. (8) Measures of at least one key outcome were obtained from more than 85% of subjects initially assigned to groups. (9) Results were presented for all subjects who received treatment or were assigned to the control group, or when this could not be done, data for at least one key outcome were analysed by “intention-to-treat”. (10) Results of statistical comparisons between groups were reported for at least one key outcome. (11) The study provides point and variability measures for at least one key outcome.

### 3.2. Assistant Robots

Social assistant robots (SARs) are robotic devices designed to provide support, companionship, and assistance to people in various environments, especially those who may need special care or additional companionship. These robots are designed to perform a variety of tasks and functions that aim to improve the quality of life of people, especially those who face physical, emotional, or cognitive challenges [40]. Some of the typical functions of these robots are companionship to reduce loneliness and isolation, physical care, medication reminders and follow-up, therapy and entertainment, assistance in daily living or communication.

**Figure 2:**
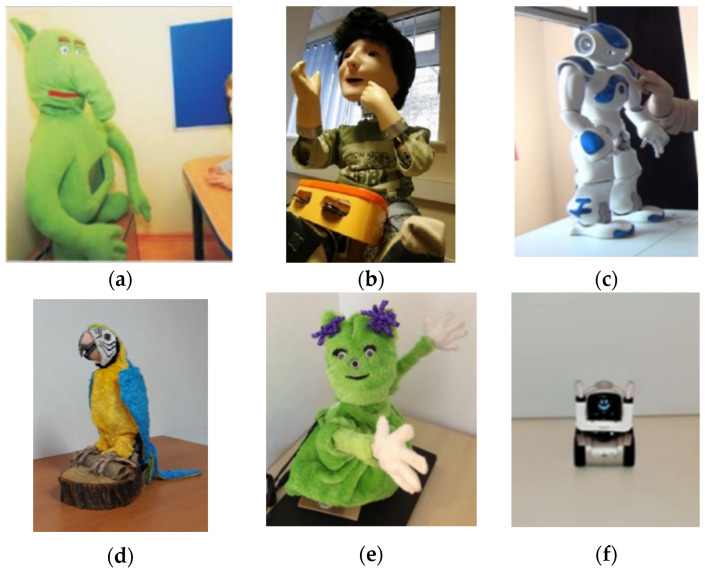
Assistant robots—assistants described in the research: (**a**) Probo [37]; (**b**) KASPAR [52]; (**c**) NAO [53]; (**d**) RoboParrot [40]; (**e**) Charlie [38]; (**f**) Cozmo [56].

Of the functions of SARs, for this review, only the functions of the robot have been taken into account within therapies and training, which are often used in occupational therapy, play therapy, and entertainment activities. SARs can vary in size and complexity, from small, simple devices to more advanced robots with artificial intelligence capabilities and sensors that allow them to adapt to the needs and preferences of people they help. They aim to improve the quality of life of people, promote autonomy, and reduce the burden on caregivers and family members in long-term care situations. Figure 2 shows the different robots used in the selected papers. In the following, these robots are described in the context of the papers in which they are used.

#### 3.2.1. Probo

The robot Probo (Figure 2a) has a fully actuated head capable of displaying facial expressions, which means that the robot can be used to express emotions with 20 degrees of freedom (DOF) in his face. In the study by Pop et al. [37] the robot is always controlled by an operator in a *Wizard of Oz*-like configuration, allowing an instant adaptation to unexpected behaviours–reactions of the participants. The *Wizard of Oz* experiment is a technique used in the field of human–computer interaction, in which subjects interact with a computer system they believe to be independent but which is actually controlled completely or partly by a human being [57]. This means that the robot does not act autonomously; that is, it does not make decisions on its own to direct the therapy, because it is controlled by a person with a PC or tablet. In Pop et al. [37], a lip synchronisation module allows the lips to move according to the voice, which consists of a pre-recorded neutral male voice. In addition, it has a soft and huggable touch, looks like a stuffed elephant, and is easy to wear.

#### 3.2.2. KASPAR

KASPAR (Figure 2b), as described in [52], is a 60 cm tall minimally expressive humanoid robot, developed by researchers from the Adaptive Systems Research Group at the University of Hertfordshire, which sits in a seated position (like a child). It has been used to study various forms of human–robot interaction and communicates primarily with people through gestures, facial expressions, and speech (playing back pre-recorded messages). The robot has 14 degrees of freedom (8 DOF in the head–neck and 6 in the arms). The face is a silicone rubber mask supported by an aluminium frame. It has 2 DOF in the eyes, eyelids that can open and close, and a mouth capable of opening and smiling.

#### 3.2.3. NAO

NAO (Figure 2c) is a humanoid robot developed by Aldebaran Robotics, France, 58 cm tall and 5 kg heavy with 25 degrees of freedom. NAO moves with agility, with an inertial navigation device to maintain stability, and can detect and avoid obstacles using two pairs of ultrasonic transmitters and receivers, which enable precise movement. NAO is balanced by four pressure sensors that control the corresponding centre of pressure on each foot. It has four speakers and a speech recognition and analysis system, which allow it to listen, speak, and perform spatial acoustic positioning, and two high-definition CMOS cameras that enable forward vision; such powerful hardware endows NAO with a high degree of artificial intelligence [58].

#### 3.2.4. RoboParrot

RoboParrot (Figure 2d) is based on a Hasbro Toy Company toy, modified and with some hardware added to control the robot. According to the investigation by Dehkordi et al. [40], this hardware provides the communication between the robot and the computer. The RoboParrot robot is composed of various sensors, mechanisms, and software that allow it to interact with children and perform assessments for autism. It has sensors such as a microphone, infrared (IR) sensor, and Hall effect sensor in the beak, which allows the robot to detect the proximity of a hand on its head and beak. To perform movements, the robot has two main mechanical motors that control the movement of the body and head of the robot, where the body motor controls the movement of the wings, legs, and neck, and the head motor controls the movements of the eyes and the beak of the RoboParrot. All motors and sensors are monitored through this controller.

To interact with children, the robot is able to close and open its eyelids, beak, and wings. It can also move its body in three directions and its neck forward and backward or left and right, as well as using the speaker to make sounds and the microphone to pick up sounds. The study by Dehkordi et al. [40] developed a Graphical User Interface (GUI) module that provides tools for an operator to control the robot and a voice modulation module that filters and changes the operator’s voice so that it more closely resembles the voice of a parrot. The control system and user interface have been designed so that the operator can see an autistic child through the camera and interact with him/her verbally or by moving the robot.

#### 3.2.5. Charlie

Charlie (Figure 2e) is the robot used by Boccanfuso et al. [38]. The robot’s hardware includes six servos, three pan–tilt platforms, an eight-channel servo controller, a consumer-grade webcam, and a lithium-polymer battery. The arms and head are mounted on a pan–tilt platform using large metal snap fasteners, with each platform controlled by two servos. The resulting two degrees of freedom in the robot’s arms allow a wide range of hand poses, while the two degrees of freedom in the head allow the robot to effectively track the face of each participant. The fundamental structure of the robot has a kinematically simplistic design with few degrees of freedom. This hardware setup allows the robot to imitate a wide range of hand poses, effectively track the face of each participant, and perform autonomous hand/arm motions during interactive play. Additionally, the robot’s body is padded for safety, covered with a nonthreatening fur-like material, and equipped with LEDs in the hands for positive feedback and a speaker for auditory instructions and positive feedback. The main role that it plays is free play in the early social development of the child, for which it has integrated detachable arms and head, as well as a base that can be attached to a table. The robot’s appearance resembles a toy to attract the attention of young children with ASD and to avoid, as far as possible, being intimidating.

#### 3.2.6. Cozmo

Cozmo (Figure 2f) is a robot, powered by a smartphone app, used in therapy for children with ASD. This robot is shaped like a crane truck, which is attractive for children. It can be moved by wheels and has sensors to detect commands, which implies the presence of hardware components capable of detecting specific input signals or patterns (instrument that detects and measures physical properties) and associated software to interpret these signals as user commands. Taking advantage of these characteristics, Davide et al. [53] work with a series of interactive cubes that can be used in different ways in therapies for children with ASD.

### 3.3. Therapies and Activities

The aim of this article is not only to know the tools (different robots, computers, etc.) used in therapies with children with ASD, but also to know what these therapies are like. For this reason, case studies, reviews, RCTs, etc., that did not adequately describe the type of therapy and activities used were discarded from the review, as indicated above, developed in the Section 2.

Just as different robots were observed in the reviews, the different therapies that the children performed were also classified. It is necessary to consider that the autism spectrum is very wide and that each child, due to his age and how autism affects him, must receive a different therapy. Autism is a developmental disorder that affects communication, social interaction, or skills and behaviour. For this, each therapy has a function or approach according to the needs of each child. Regarding this review, with assisted robots, the usual therapies are structured games or activities that encourage work in a specific area (paying attention, mistrust, emotions, etc.). Regarding these therapies, the key features of these robots are summarised in Table 4.

#### 3.3.1. Social Stories

Social stories (SS) are short and educational stories for children. These stories show realistic pictures that are intended to help an autistic child better understand and/or navigate his world [59]. For this type of therapy, it is essential that the robot can emit sound, i.e., it has a loudspeaker. In addition, it is also useful to accompany the voice with images or pictograms, which can be obtained from a tablet or printed on paper. Given the characteristics of all the robots, Probo is the robot best suited to this type of activity, as it has a loudspeaker to tell the story, a tablet to reproduce images, and can make gestures to capture the child’s attention. Other robots such as KASPAR or NAO can also be used, but an external tablet must be added.

This type of therapy starts with an introduction; in the case of the study by Davide et al. [56], it starts with “The story starts now” or “Let’s listen to the story”, etc. Then, taking into account the child’s attention, three global questions are asked immediately after the end of the story. Then, Probo, the robot, creates the experimental task by giving the necessary clues to the participant from the story. Afterward, the task clues are put into practice in a natural and appropriate everyday environment, offering help to the child to give the correct answers. Finally, feedback is given to the child.

#### 3.3.2. Imitation Gesture

A variant of social stories is the imitation gesture. This type of therapy uses a robot to try to get children to imitate his gestures. It is a very complex therapy and requires robots with a high number of degrees of freedom, because it is necessary to be able to reproduce the gesture as well as possible. Robots that are best suited to this type of therapy are NAO and KASPAR, as they have a great capacity to reproduce gestures by means of their joints (arm, neck, etc.). In the case of NAO, a wide range of movements can be achieved; not only movements with the torso and arms, but also movements with the lower limbs; this robot, in addition to performing gestures, can be programmed to provide feedback and motivate the child, thus maintaining the child’s attention in a better way.

In [28], the authors implemented 20 commonly used gestures in daily life. In this case, a virtual robot is used, not a physical robot, a computer simulation model that mimics the behaviour of the NAO robot. These gestures are used in everyday life as a greeting or to show emotions. In addition, audio clips are added that describe each gesture. In each of the clips, the gesture and the corresponding speech start at the same time. In this way, the children are able to watch videos of gestures while listening to the audio clips.

#### 3.3.3. Games Therapies

This therapy uses structured play to promote social, emotional, and cognitive development. Play therapists work on social interaction, imagination, and creativity. Throughout the review, many structured games were observed to work in different areas, such as memory, social skills or patience, objects or living beings, colours, etc. [60]. Some of these structured game therapies, such as series games, although simple, manage to work in several areas, where the child works to understand basic concepts and overcome rigidity (difficulty in following orders) [61].

In this case, a minimum requirement is that the robot can reproduce sound (to give the commands). But to perform the therapy dynamically and to keep the child’s attention, feedback is necessary. Moreover, as there is a great variety of structured games, the necessary capabilities of each robot vary, from only needing sound to needing LEDs, movements, image capturing, etc., with NAO being the most complete robot in this sense.

Various studies use this kind of therapy, such as Wainer et al. [52], where players faced each other during each session, and to play the video game cooperatively on the horizontal screen, players had to synchronise and coordinate their actions properly; the game did not register the actions of a single player if they were not performed at the same time and in the same way as those of the other player [11].

#### 3.3.4. Joint Attention

In this type of therapy, children attend to the same thing and they are mutually aware they are doing so, responding to questions. For autism, it is very difficult for children to do it because they lose interest quickly. Joint attention is the focus of many early intervention studies on autism. Collaborative attention refers to the development of a style of attention that involves cooperation and collaboration with others, as well as the development of specific skills that involve sharing ideas with others. This involves the development of skills such as pointing and showing objects and establishing a relationship between people [62].

As in games therapies, this type of therapy is very varied. Mainly, the voice or sound of the robot is required to capture the child’s attention, but any type of stimulus can be introduced (sound, image, gesture, lights, etc.); depending on the level of autism, several could be introduced at the same time. However, any of the previously described robots could be used, taking into account how we want to focus the therapy. It is also important to consider that, for this kind of therapy, NAO, KASPAR, and Cozmo are the robots with the best characteristics.

Some studies propose a simple task based on instruction, response, and consequence, such as David et al. [53]. Other articles used a complex system that includes a humanoid robot that provides joint attention signals. For example, in [50], a humanoid robot, two target monitors that could be activated contingently when children looked at them, an attention-tracking subsystem consisting of four spatially distributed cameras, and a controller of these elements reporting results and responding in real time, are used to work with this type of therapy.

#### 3.3.5. Learning Distrust and Deception

This activity or set of activities is very complex. They are included in social skills therapies. It is about teaching children to be wary of possible situations where they can be fooled. For this, it is necessary that children learn to identify deception. It is reasonable to expect children to gain confidence (acquire the skill of distrust and deception), because their ability to understand their environment increases with age. This expansion shows people who have nothing good to say about children or who do not want children. A trust option helps children avoid being misled by information from those people and also ensures that they receive authentic information that meets their developmental and life needs [63].

Zhang et al. [55] show that children with ADS can learn, through activities with the humanoid robot NAO, the distrust tasks. The tasks of mistrust and deception require a deep understanding and manipulation of the mental states of others [63]. Children typically learn these rules through interpersonal interactions and by interpreting various verbal and non-verbal social cues from past experiences [55]. Overcoming these tasks involves the ability to perceive relativity and cope with more advanced and complex information, ultimately leading to the development of cognitive skills and social understanding. This type of therapy is hardly ever developed with robots, as it is very complex. In these studies, NAO was used because it is a humanoid robot (with a human-like appearance) and can perform complete movements and reproduce sound [55].

## 4. Discussion

**Figure 3:**
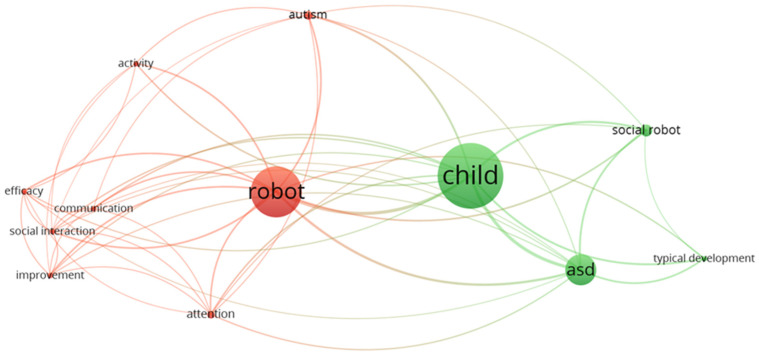
Keyword cluster analysis (in colours). VOSViewer analysed the keywords of the selected papers that were used together.

The following shows a map based on text data from a reference manager (.RIS) file that contains all the articles evaluated for this review, using the VOSViewer tool 1.6.20 (see Figure 3). Each colour represents a cluster, curved lines are relationships associated with keywords, and density is related to occurrences. The words autism and ASD refer to the same term and are related to two of the clusters (red and green). The red cluster contains words related to the therapies and outcomes of the articles (efficacy, social interaction, attention, etc.). The main clusters are discussed below.

The interest of this review was to explore the use of robots with children with ASD at early ages (0–6 years); however, most of the studies conducted so far are with children outside the early ages. We included studies with mainly early-age children with ASD, and papers involving children under the age of 10 years were also accepted if the sample included early-age children (aged 6 years or younger). It was observed that in those articles where the samples contained a larger number of children, they usually included children older than the age of six. Papers in which only younger children are included have a smaller sample number.

On the other hand, SARs can have various forms. They are usually classified as humanoid, animal-like, or other (alternative forms such as Cozmo, which looks like a truck). Throughout this review, research was found with all the types of robots mentioned (Figure 2). Then, it was observed that, with humanoid robots themselves, the most widely used was NAO, which is used in approximately 30% of the research on social robots for autistic children [58]. Of the studies reviewed for this paper, the NAO robot was used in 44.44% of the cases. This is in line with other studies and is reasonable, as can be seen in Table 4, because humanoid robots tend to have better capabilities (higher number of degrees of freedom, joint movements, displacements, etc.).

Additionally, the therapies used are varied. Each of the therapies focuses on a different aspect, so they were adapted to the problem they wanted to solve. In these therapies, the robots act as assistants to the therapists, which means that they never replace the person, but are used to help carry out the therapy. Firstly, the objective is to improve the social skills of children with ASD through robotic interventions. Both Pop et al. [37] and Boccanfuso et al. [38], which use animal-like robots and alternative robots, respectively, focused their therapeutic efforts on this aspect, using different robots to achieve positive results. Another crucial similarity lies in the use of robots as facilitators of social interactions. Dehkordi et al. [40] and Wainer et al. [52] employed robots, with animal and humanoid looks, respectively, to encourage interaction, specifically adapting to the individual preferences of children, highlighting the ability of robots to influence social dynamics.

The application of structured games also emerges as a similarity among the studies. Boccanfuso et al. [38] and Zheng et al. [50] used therapeutic games with robots to improve social and cognitive skills. David et al. [53] used therapeutic games sessions with the NAO robot, demonstrating the versatility of this strategy in therapeutic settings. Joint attention, a vital component in social development, was addressed in several studies. David et al. [53] emphasised improvements in joint attention skills through therapy with the NAO robot. So et al. [54] also addressed joint attention using gestures and robotic narratives, emphasising the importance of this aspect in interventions.

Furthermore, the evaluation of results through standardised measurements is a common practice. Boccanfuso et al. [38] and Davide et al. [56] standardised scales and questionnaires to assess the progress of children, providing a quantitative approach to therapeutic evaluation. Zhang et al. [55] and Zheng et al. [50] used specific tests and measurements to assess the results, helping to improve objectivity in the evaluation of the impact of interventions.

Finally, consideration of the individual preferences of children is highlighted in various studies. Dehkordi et al. [40] and Davide et al. [56] use robotic therapy taking into account individual preferences, recognising the importance of personalising interventions for each child. Moreover, in the study by Wainer et al. [52], the interaction of the KASPAR robot with a focus on preferences and affection underscores the relevance of this factor in designing effective interventions for ASD. All these studies indicate common patterns in the application of robotics in ASD interventions, highlighting the importance of improving social skills and adapting to the individual needs of children affected by this disorder.

In the case of Pop et al. [37], it was found that the use of a social robot to implement social story interventions was more effective in improving independence in the expression of social skills compared to the use of a computer screen. Wainer et al. [52] noted that, although there was no quantitative increase in positive affect expression when interacting with the KASPAR robot compared to peer interaction, longer durations and more positive affect-showing cases were observed while children looked at other peers.

In the case of robots similar to animals or other, the intervention in [40] revealed that a parrot-like robot was successful in encouraging children to interact according to their individual preferences, aligned with the verbal, functional, and perceptual functionalities of the robot; and Boccanfuso et al. [38], who reported a significant increase in social interaction skills, as well as improvements in specific areas as reported by caregivers, also offered results that improved the skills of children with ASD.

David et al. [53] found that, through the use of the NAO robot, children maintained their interest throughout the sessions, showed high adherence to treatment, and experienced improvements in their joint attention skills. Also, So et al. [54], using the NAO robot, found that children with ASD in the intervention group were more likely to produce accurate or appropriate gestures during training sessions, and these positive results were maintained in subsequent tests.

Zhang et al. [55] explored the development of mistrust in children with ASD, noting the possibility of applying these learnings to real-world situations. However, Zheng et al. [50] cautioned that mixed patterns of improvement and worsening suggest that robotic interventions may not be universally appropriate for all children on the autism spectrum.

Finally, Davide et al. [56] concluded that combined therapies, which incorporate iterations, were more effective than individual therapies as assessed by the ADOS test and ESCS. Taken together, these findings suggest that the use of robots in therapy for children with ASD may have beneficial impacts in a number of areas, although a more complete understanding of the suitability and long-term benefits of these robotic interventions is required.

Autism is a very broad spectrum, so comparing groups can be very complicated. Table 2 shows the papers analysed; the analysis of the various works on the different scenarios is shown, such as the performance or effectiveness of the therapies, taking into account the different depths (or levels) of autism (mild, moderate, and profound), the difference between acting alone or with other children in a therapy, and also the differences between conventional therapy (without robots) and therapy with robots in each case. After the analysis of all the results, two main ways of analysing the effectiveness of the therapies were observed: simple or complex parameters. Simple parameters are those that do not require a specific test such as holding the gaze, counting the number of times the child is able to ask for help or ask for something, the number of correct answers, etc. Complex analyses were also performed, such as the ADOS-2 task, VABS-II, SCQ, EVT-2, ESCS or MIS to analyse the results of robot-assisted therapies.

In the cases of the [37,50,52,53,54,55] authors, they did not use a specific scale, but each author in these cases focused on a particular measure. In [37], the authors opted for a general assessment of social skills. On the other hand, Wainer et al. [52] made observations about the duration and frequency of positive affective expressions, and David et al. [53] about behaviours related to joint attention, while So et al. [54] focused on the accurate production of intransitive gestures. Zhang et al. [55] performed specific tasks related to distrust and disappointment, and Zheng et al. [50] measured the intervention levels and success rates in the robotic intervention session.

Other works used the DSM-IV-TR scale [28,64] to rate specific behaviours, such as eye contact and interaction preferences [40]. On the contrary, in [38], the authors deployed multiple scales, including VABS-II, MLSUD, UIA, MIS, and EVT2, addressing areas such as social skills, language development, and imitation, and in [56], the ADOS and ESCS scales were used to assess social skills, behavioural requests, and joint attention.

Ethical considerations play a crucial role in the design and implementation of social and emotional robots, especially in therapeutic contexts involving individuals with ASD. Involving stakeholders such as therapists, parents, and individuals with ASD ensures that the design and implementation process considers diverse perspectives and concerns [43]. Ensuring that robots are safe to interact with children and therapists is essential, thereby building trust among users through transparency, reliability, and clear communication about the robot’s capabilities and limitations. Design decisions, such as making robots resemble humans (as in the case of KASPAR [52]) or animals (as in the case of RoboParrot [37,40]), can influence user perceptions and emotional attachment to robots. The ethical considerations here revolve around whether such design choices are appropriate and whether they might inadvertently lead to unrealistic expectations or unhealthy attachments. Ethical practice requires that interventions, including those facilitated by social robots, be effective in achieving therapeutic goals. Regular evaluation is necessary to ensure that robot use contributes positively to the well-being and development of children with ASD, rather than simply being a gimmick or distraction [43]. Although robots may not inherently pose privacy risks, their integration into therapeutic settings may introduce new privacy considerations. In addition to obtaining written consent by informing the parents or legal guardians of the children participating in the study, as specified in [56], it is important that the robotic system does not violate the contract terms. In addition, understanding how users perceive robots and addressing any biases or misunderstandings is crucial to fostering positive and productive interactions. By carefully and proactively addressing these ethical considerations, researchers and developers can create social and emotional robots that enhance therapy for individuals with ASD, while respecting their rights, dignity, and well-being. The ongoing dialogue and collaboration with stakeholders remains essential to navigate the complex ethical landscape of social robotics in a responsible way.

A key consideration in various studies is the recognition of the individual preferences of children. The adaptation of robotic therapy to account for these preferences is acknowledged as important, highlighting the importance of personalising interventions for each child. This emphasis on individualisation is also reflected in the diversity of ASD, where differences between mild and moderate autistic children, those with profound autism, the presence or absence of other peers in therapies, and the participation of robots are considered.

The robotic systems used in the reviewed studies addressed a variety of specific deficits in social relations, communication, comprehension, flexibility in reasoning, etc. First, they focused on improving fundamental social skills, such as eye contact, the ability to ask questions, and appropriate greetings. These skills are critical to establish and maintain meaningful relationships with others, but they are areas in which children with ASD often struggle. These aspects can also be considered as possible measures to evaluate the effectiveness of the therapy, since it is possible to measure how long the child holds the gaze or how many times the child points to an object, etc.

In addition, specific difficulties in non-verbal communication, such as understanding and producing gestures, were addressed [54]. Robotic systems were used to teach children to recognise and use gestures appropriately as part of their social interaction. The participation in social and cooperative interactions was also promoted, both with the robot and among the children themselves, encouraging collaborative play and teamwork. In terms of flexibility in reasoning, robotic systems provided opportunities to develop problem-solving skills and adaptation to different situations. For example, Zhang et al. [55] even explored the understanding of complex social concepts, such as deception and mistrust, as a means of improving cognitive flexibility and understanding of social rules.

The pursuit of the generalisation of skills acquired through interaction with robotic systems was also key. This involved ensuring that the social and communicative skills learnt with the robot could be transferred to situations of interaction with real people. In this way, children with ASD could effectively apply their skills in the real world, promoting their social participation and integration in social settings.

Several limitations and difficulties were identified in the reviewed studies. A notable limitation was the small sample size of some of the studies shown in Table 2 (such as Wainer et al. [52], David et al. [53], Boccanfuso et al. [38]), which ranged from five to eight participants in EG, which could lead to the influence of individual variations. Furthermore, it was noted that most of the studies were conducted for limited numbers of therapies or short periods of time; although this may not seem like a limitation in principle, extending the research would yield data on the effectiveness of using robotic systems in the long term and whether the child actually loses attention over time.

Some earlier studies, such as that of So et al. [54], did not control children’s motor and memory skills, which could influence their ability to learn gestures. Another limitation was the uncertainty of whether the children in these studies understood the meaning of the gestures they imitated. This lack of understanding could affect the appropriateness of the gestures produced and their generalisability to new environments. In addition, the absence of assessment of the children’s mood was also identified as a limitation, as mood can influence responsiveness and interaction.

Furthermore, the limited capabilities of some robots, such as the restricted field of view of the camera, made it difficult to assess certain behavioural traits (Table 4). For example, the use of a parrot-like robot [40] or a toy truck [56] limited the range of interactions, as it lacks features such as pointing or large physical movements, which are important for sensing and therapy approaches. 

In terms of limitations, it is important to consider that these could affect the overall conclusions drawn from the research by potentially limiting the scope and precision of the results. It is important to address these limitations in future studies to ensure the robustness and reliability of the research results. There were also challenges to be solved, such as ensuring the flexibility and adaptability of robotic interventions to address the changing needs of children with ASD over time. The ability to customise interventions and adjust them according to individual progress is crucial to maximise the therapeutic benefits.

In terms of results, the general effects of the introduction of robots in therapies were positive. Studies showed improvements in parameters such as maintaining gaze, the number of correct answers, and learning behaviour. Although there are differences between studies due to the broad spectrum of autism, the positive impact on children’s behaviour, attention, and learning was evident.

In summary, the use of robots, in general, improves children’s behaviour in the short term. In future work, the use of robots should be tested in the longer term, which means a total period during which the research will be carried out, from the beginning of the project to the completion of the final report, with a longer duration in time, as most of the articles reviewed in this paper have a duration of a few months (approximately 6 weeks to 3–4 months), except the study by Wainer et al. [52], which has a duration of one year. As far as research is concerned, it is noted that hardly any work has been done on children exclusively at an early age. Also, it was observed that the ratio of females to males was much lower; therefore, in those articles reporting the number of males, they showed a low female population.

## 5. Conclusions and Future Work

This review aimed to investigate the application of robots in therapy for children with ASD at the early ages of 0–6 years. When delays are observed in any of the deficits they may present (social, language, communication, etc.), it is necessary to intervene to prevent them from becoming chronic and established. Most of the studies conducted so far involved children outside of this early age range. Regarding SARs, they come in various forms, including humanoid, animal-like, or others. This review found research that involved all the types of robots mentioned. The NAO humanoid robot was the most complete and widely used.

Therapeutic approaches varied from study to study, each focusing on specific aspects to address distinct challenges. In particular, emphasis was placed on the social skills of children with ASD through robotic interventions. Studies directed their efforts toward improving social skills, using different robots to achieve positive outcomes adapting to individual preferences. Structured games have emerged as a common element in various interventions, as observed in studies where robot-based games aim to enhance social and cognitive skills. Additionally, games are used in therapeutic sessions with NAO robots, showcasing the versatility of this strategy in therapeutic contexts. Finally, multiple studies address the crucial aspect of joint attention in social development, emphasising improvements in joint attention skills through robot therapy and approaches using gestures and robotic narratives. The key implications and challenges identified in the comparison of the different robotic interventions are to know what work methodology has been used, the duration of the therapies, the involvement of the professionals, and the possibility of knowing the cost of its implementation.

The field of robotics applied to autism therapies is continually evolving, and there are several promising areas for future research. One potential direction includes the development of robots specifically tailored to address the individual needs of children with different levels of autism. This involves customising the robot’s appearance, behaviour, and capabilities to maximise the effectiveness of therapy. Furthermore, the integration of emerging technologies, such as virtual and augmented reality, could be explored to enhance the therapeutic experience. Investigating how artificial intelligence and machine learning can improve the adaptability of robots to the changing needs of children with autism also represents an intriguing area of research. Another direction could focus on collaboration between robots and human therapists, exploring how robots can act as assistants, helping in the implementation of therapeutic programmes and collecting data to assess the child’s progress.

Finally, the application of robots in formal educational settings, such as classrooms, could also be studied to support the academic and social development of children with autism. In addition, robot-based remote monitoring and telehealth can be investigated to provide remote and personalised therapy. Exploring ethical and social issues related to the public acceptance of robots in therapeutic settings, as well as data privacy, is essential. Long-term evaluation of the impact of robotic interventions on the development and well-being of children with autism should also be considered.

## Figures and Tables

**Table 1:** Exclusion/inclusion criteria.

Inclusion	Exclusion
The sample contains young children (aged six years or less).	If the sample contains children older than 10 years, even if it contains young children, or does not contain children younger than 6 years.
Technological tools are used (robots, computers, tablets, etc.)	The sample is not clearly specified: neither the mean age or deviation nor the number of children are shown.
The type of therapy is specified (time and number of sessions, type, etc.)	The robot used is not specified.
The study contained samples with five or more children.	If the purpose of the use of the robot is not to perform therapy.
	The methodological quality is lower than 4 points on the PEDRo scale.

**Table 4:** Key features of robots needed in therapies.

	Probo	KASPAR	NAO	RoboParrot	Charlie	Cozmo
Touch sensor		X	X			
Image capture		X	X	X	X	X
Speaker	X	X	X	X	X	X
Microphone		X	X			
LEDs			X			X
Tablet or smart phone	X					X
Cable connection	X		X	X	X	
WiFi connection		X	X	X		X
Joint movement	X	X	X	X	X	X
Displacement			X			X
Degrees of freedom	20	14	25	-	-	-

Boxes marked with an X indicate that the robot meets the requirement. The degrees of freedom box indicates with a number the degrees of freedom they have; if not known, it is indicated with a -. Joint movement refers to having joints such as the elbow, shoulder, neck, and being able to perform movements or actions such as grasping objects, turning the head to be able to follow the gaze, etc. Displacement refers to the ability to move from one place to another; i.e., by moving leg joints (hip, knee, ankle) or other mechanisms (wheel, rails, etc.) the robot is able to change its spatial position.

## Data Availability

No new data were created or analyzed in this study. Data sharing is not applicable to this article.
